# Clinical and Preclinical Systematic Review of Astragalus Membranaceus for Viral Myocarditis

**DOI:** 10.1155/2020/1560353

**Published:** 2020-11-02

**Authors:** Qun Zheng, Zhuang Zhuang, Zi-Hao Wang, Li-Hui Deng, Wang-Jun Jin, Zi-Jun Huang, Guo-Qing Zheng, Yan Wang

**Affiliations:** ^1^Department of Rheumatism and Immunity, The Second Affiliated Hospital and Yuying Children's Hospital of Wenzhou Medical University, Wenzhou, China; ^2^Department of Cardiology, The Second Affiliated Hospital and Yuying Children's Hospital of Wenzhou Medical University, Wenzhou, China; ^3^Department of Neurology, The Second Affiliated Hospital and Yuying Children's Hospital of Wenzhou Medical University, Wenzhou, China

## Abstract

Astragalus membranaceus (AM) is a traditional Chinese medicine, which possesses a variety of biological activities in the cardiovascular systems. We conducted a clinical and preclinical systematic review of 28 randomized clinical control studies with 2522 participants and 16 animal studies with 634 animals to evaluate the efficacy, safety, and possible mechanisms of AM for viral myocarditis (VM). The search strategies were performed in 7 databases from inception to January 2020. Application of the Cochrane Collaboration's tool 7-item checklist, SYRCLE's tool 10-item checklist, and Rev-Man 5.3 software to analyze the risk of bias of studies and data. The results show the score of clinical study quality ranged from 3 to 7 points with an average of 3.32, and the score of animal study quality ranged from 2 to 5 points with an average of 3. In clinical study, AM significantly reduced serum myocardial enzymes and cardiac troponin I levels and improved the clinical treatment efficiency in VM patients compared with the control group (*P* < 0.05). There was no significant difference in the incidence of adverse reactions (*P* > 0.05). Significant increase of the survival rate and decrease of the cardiac cardiology score, cardiac enzymes, and cardiac troponin I were compared with the placebo group in animal studies (*P* < 0.05). The possible mechanisms of AM are largely through antivirus and antivirus receptors, anti-inflammatory, antioxidation, antiapoptotic, antifibrosis, and reducing cardiac calcium load. In conclusion, the findings suggested that AM is a cardioprotection candidate drug for VM.

## 1. Introduction

Viral myocarditis (VM) is defined as the inflammatory disease that injured the muscular tissues of the heart, which refers to the pathological lesion including focal or diffuse myocardial cell degeneration and necrosis, interstitial inflammatory cell infiltration, and fibrous exudation caused by viruses [1]. The acute inflammation may develop into subacute and chronic gradually to tissue remodeling, fibrosis, and loss of myocardium architecture and contractile function finally leading the myocarditis of dilated cardiomyopathy (DCM) [2]. It may cause acute heart failure (AHF) and sudden death which is counted at 10% of total sudden death [3]. In addition, the global incidence of myocarditis estimates about 10 to 20 cases per 100 000 of the population, and with the improvement of diagnosis, the prevalence and incidence expected 46% increases in 2030 [4]. According to the causal pathophysiology and clinical symptom of VM, three main treatments including conventional medical treatment, immunomodulatory therapy, and immunosuppressive therapy are used [5]. However, establishing the potential benefits of immunomodulators and antiviral therapy is currently at the preliminary research stage [6]. Although great progress such as intra-aortic balloon pump, ventricular assist device, or extracorporeal membrane oxygenation has been reached in the treatment of cardiac end-point events, the more important goal is to prevent or delay their progress and prevent complications in VM patients [7]. Thus, how to effectively treat VM and prevent AHF has attracted more and more attention to the world.

Astragalus membranaceus (AM) is a famous Qi-tonifying and immunomodulating herb in traditional Chinese medicine [8]. The main components of AM include flavonoids, saponins, polysaccharides, amino acids, and trace elements [9]. It has been widely used as a natural immunomodulator in the treatment of many immune diseases including nephritis [10], immune reaction of cancer [11], and systemic lupus erythematosus [12], and it also showed efficacy in protecting the myocardium in cardiovascular diseases [13]. In recent years, clinical and basic studies have reported the positive therapeutic effect of AM for VM. However, the scattered clinical evidence and uncertain mechanisms limited the application of AM in the clinic. Therefore, in the present study, we are aimed at comprehensively and systematically evaluating the efficacy, safety, and possible mechanisms of AM for VM from clinical and preclinical aspects.

## 2. Methods

### 2.1. Data Sources and Search Strategies

A systematic literature search for the true randomized and controlled studies (RCTs) [14] and animal experimental studies of AM for VM was carried out using PUBMED, EMBASE, Web of Science, Cochrane library, China National Knowledge Infrastructure, Wanfang, and VIP database. All search strategies were performed from inception to January 2020 with the search keyword: “Astragalus” AND “Viral myocardial”. Besides, reference lists from the resulting publications and reviews were searched carefully for the potential eligible studies.

### 2.2. Eligibility Criteria

Two authors selected the studies independently by screening the abstracts and full texts according to the eligibility criteria. Clinical research was included if it met the following criteria: (1) true RCTs of AM for VM with the accepted methodology for randomization: the study which randomized sequence was generated by randomized sequence, calculator, or computer random number generator was included preferentially; coin-tossing or drawing straws in the absence of the participant to decide which group the next participant would be assigned to were also considered eligible randomization techniques [14]; (2) the selected participant should match VM diagnose [2, 15, 16]; (3) the treatment group involved AM as monotherapy or plus basic treatment with unrestricted dosage, formulation, route of administration, and administration time, and the control group received basic treatment, placebo, basic treatment plus placebo, or no treatment as treatment; (4) the primary outcome measures were mortality or survival rate and/or the main cardiovascular events and/or myocardial enzyme and/or cardiac troponin level and/or the heart function index of ultrasonic cardiogram. We adopted the efficiency of clinical therapy and adverse reaction as the second outcome measures. Animal research was included if it met the following criteria: (1) controlled studies assessing the *in vivo* administration of AM for VM established by various ways were included; (2) the treatment group involved AM as monotherapy with unrestricted dosage, formulation, route of administration, and administration time, and the control group received placebo or no treatment as treatment; (3) the primary outcome measures were mortality and/or survival rate and/or cardiac pathology and/or myocardial enzyme and cardiac troponin level and/or the heart function index of ultrasonic cardiogram, while the second outcome measures were cardioprotective mechanisms of AM. Exclusion criteria of the clinical and animal researches were as follows: (1) not true RCT study or animal study (in vitro studies, case reports, clinical trials with unaccepted methodology for randomization, reviews, abstracts, comments, and editorials); (2) compare with other Chinese herbals; (3) treatment with AM conjunction with other compounds in animal study; (4) duplicate publications; (5) no any primary outcome indicator were involved or incomplete date; (6) no control group; (7) not VM model.

### 2.3. Data Extraction

The information were extracted from included studies by two independent authors using a predefined form. Clinical study extracted author, year, the number of participants, ratio of male and female, the therapeutic regimen for treatment and control groups, adverse reaction, and outcome index from each study. Animal study extracted author, years, detail of animals participating in the experiment, the method to induce the model, the therapeutic regimen for treatment and control groups, and outcome index. Only the outcome data of the highest dose group and peak time point group were included. The graph data were measured by Photoshop when the results were only rendered by graphics, and the response was not received from the corresponding authors.

### 2.4. Quality Estimation of Included Studies

The risk of bias tool recommended by Cochrane Collaboration [17] (The Cochrane Collaboration.http://www.cochrane-handbook.org. (Accessed December 25, 2014)) and SYRCLE's risk of bias tool [18] was adopted separately to estimate the quality of included clinical and animal studies. Disagreements in the process of selecting studies, extracting data, and assessing the quality of studies were resolved by consensus or arbitration by the correspondence authors.

### 2.5. Statistical Analysis

The RevMan 5.3 was used to dispose the data of detailed outcome where possible; otherwise, the system assessment was adopted. Random (*I*^2^ > 50%) or fixed-effects model (*I*^2^ < 50%) was selected according to the results of heterogeneity estimated by using the Cochrane *Q*-statistic test and the *I*^2^-statistic test. The effect sizes of continuous variable were estimated by utilizing standard mean difference (SMD) with 95% confidence interval (CI), and the effect sizes of bivariate were estimated by utilizing odds ratio (OR) with 95% CI. The Forest plot was used to present meta-analysis results, and the funnel plot was used to assess reported bias when a single index included more than 12 studies. The difference between treatment and control groups was considered statistically significant when *P* < 0.05.

## 3. Results

### 3.1. Study Selection

**Figure 1:**
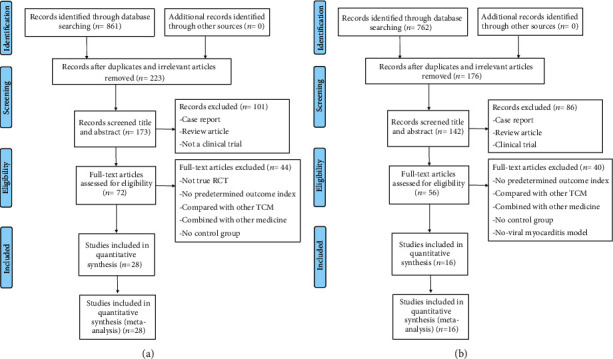
Summary of the process for identifying candidate studies. (a) Search strategy for clinical studies. (b) Search strategy for animal studies.

For clinical studies, a total of 173 studies were extracted from initially collected 861 studies after scanning the titles and abstracts. Detailed inspection was performed to remaining full-text studies; 145 studies were excluded according to the inclusion and exclusion criteria. For animal studies, a total of 142 studies were extracted from initially collected 762 studies after scanning the titles and abstracts. Detailed inspection was performed to remaining full-text studies; 126 studies were excluded according to the inclusion and exclusion criteria. Finally, 28 randomized controlled clinical trials and 16 animal studies were included. The detailed search process was shown in Figure 1.

### 3.2. Characteristics of Included Studies

#### 3.2.1. Clinical Studies

The overall characteristics of included clinical studies are generalized in Table 1. All included studies were RCTs published in Chinese from 2006 to 2019. Among them, 9 studies [19–27] are involved in adult with VM and 19 studies [28–46] in children with VM. With regard to the information of the participants in the experiment, a total of 1276 subjects were included in the intervention group, while a total of 1246 subjects in the control group. The baseline of the two groups was comparable. Twenty-one studies [19–22, 24, 26, 27, 29, 31, 33–35, 37, 39, 42–46] implemented the dose gradient of AM ranged from 5 ml•d^−1^ to 200 ml•d^−1^ by intravenous drip infusion administration. In addition, 0.5 ml•kg^−1^•d^−1^ Astragalus membranaceus injection (AI) was administered by intravenous drip infusion in 1 study [30], 1 ml•kg^−1^•d^−1^ in 1 study [36], 2 ml•kg^−1^•d^−1^ in 1 study [28], 2 g•kg^−1^•d^−1^ in 1 study [41], and 20 g•kg^−1^•d^−1^ in 1 study [25]; and the oral dosage of AM granule was adjusted according to age (2 g/d (age ≤ 2Y), 3 g/d (2Y < age ≤ 4Y), 4 g/d (4Y < age ≤ 6Y), and 8 g/d (age > 6Y)) and was reported in 2 studies [32, 38]. Except 1 study [37] reported that it only contrasted the curative effect of AI and placebo without additional treatment, the remaining 27 studies reported that the intervention group and the control group were given basic treatment (including antivirus, anti-infection, antiarrhythmia, and nourishing myocardium), and AI or granules were added to the intervention group. Detailed information on AM in each clinical study is displayed in Table 2. As for follow-up period, 18 studies [20, 22–25, 27–30, 33–38, 40, 42, 43, 45] lasted 2 weeks, 7 studies [21, 26, 31, 32, 41, 44, 46] lasted 4 weeks, 1 study [19] lasted 6 weeks, and 1 study [39] lasted 46 days. Creatine kinase (CK) was utilized as primary outcome measure in 11 studies [22, 23, 25, 28–31, 35, 37, 41, 45]; creatine kinase isoenzyme (CK-MB) in 14 studies [19, 21, 22, 25–31, 35, 37, 38, 41]; lactate dehydrogenase (LDH) in 10 studies [20, 22, 23, 25, 27, 30, 35, 38, 41, 45]; glutamic pyruvate transaminase (AST) in 7 studies [19, 25, 27, 38, 41, 42, 45]; and cardiac troponin iroponin I (cTnI) in 8 studies [21, 23, 26, 28–30, 35, 37]. Ejection fraction (EF) was utilized as a primary outcome measure in 1 study [20], and none of the included studies were involved in mortality and major cardiovascular events. The clinical efficacy of AM in the treatment of VM was utilized as a secondary outcome measure in 26 studies [19–23, 25–28, 30–46] and the adverse reactions in 10 studies [19, 26–29, 32, 33, 36, 37, 39].

#### 3.2.2. Animal Studies

The overall characteristics of the included animal study are generalized in Table 3. A total of 15 Chinese studies [47–61] and 1 English study [13] on AM for VM published between 2002 and 2017 were included. All studies are involved in 634 experimental animals. Among them, male Balb/c mice were used in 13 studies [13, 47, 49–51, 53, 54, 56–61], female Balb/c mice in 1 study [52], Balb/c mice without mentioning gender in 1 study [55], and male/female SD rats in 1 study [48]. All models of acute VM were established by intraperitoneal injection of a solution containing coxsackievirus B3 (CVB3) virus. Twelve studies [13, 48–51, 53, 56–61] implemented the dose gradient of AM ranged from 2.2 mg•kg^−1^•d^−1^ to 90 g•kg^−1^•d^−1^. In addition, 1 study [54] used the dosage of AM with 0.4 mg•d^−1^, 2 studies [52, 55] used 0.4 g•d^−1^, and 1 study [47] used 0.4 ml•g^−1^•d^−1^. Intragastric administration was adopted in 7 studies [13, 53–56, 59, 61] and intraperitoneal injection in 9 studies [47–52, 57, 58, 60]. All included studies reported that the intervention group received AM as monotherapy, while the control group was treated with the same volume of normal saline or nonfunctional liquid therapy or placebo. Detailed information of AM in each animal study is displayed in Table 4. The survival rate of animals was utilized as the primary outcome measure in 4 studies [13, 54, 57, 58], the changes of cardiac pathology or cardiac pathological score in 15 studies [13, 47–55, 57–61], cTnI in 3 studies [13, 47, 50], CK-MB in 3 studies [47, 49, 61], LDH in 2 studies [57, 61], AST in 1 study [57], and none of the included studies involved in the indexes of cardiac function under B-ultrasound. Among secondary outcome indicators for the study of mechanism, tumor necrosis factor (TNF-*α*) was reported in 4 studies [49, 53, 54, 56]; interleukin-2 (IL-2), interleukin-8 (IL-8), and interleukin-18 (IL-18) in 1 study [52]; nuclear chemokine-1 (MCP-1) in 1 study [51]; macrophage inflammatory protein-2 (MIP-2) in 1 study [50]; superoxide dismutase (SOD) and malondialdehyde (MDA) in 1 study [57]; Caveolin-3 (Cav-3) and Smad family member 3 (Smad3) in 1 study [48]; coxsackievirus and adenoviral receptor (CAR) in surface myocardium in 1 study [55]; sarco endoplasmic reticulum calcium adenosine triphosphatase (SERCA), endothelin-1 (ET-1) and the maximum binding capacity of endothelin receptor maximum binding capacity (ETR Bmax) in 1 study [13]; the replication level of CVB3 in 1 study [59]; the changes of Fas/FasL gene expression in cardiomyocytes in 1 study [60]; and the atrial natriuretic peptide (ANP) in 1 study [13].

### 3.3. Study Quality

The number of criteria met in clinical studies varied from 3/7 to 7/7 with the average of 3.32 according to the risk of bias tool recommended by Cochrane Collaboration [17] (The Cochrane Collaboration.http://www.cochrane-handbook.org. (Accessed December 25, 2014)), while the number of criteria met in animal studies varied from 2/10 to 5/10 with an average of 3 according to SYRCLE's risk of bias tool [18]. Detailed results of methodological quality of clinical and animal studies are presented, respectively, in Tables 5 and 6.

### 3.4. Effectiveness

#### 3.4.1. Outcomes of Clinical Studies

**Figure 2:**
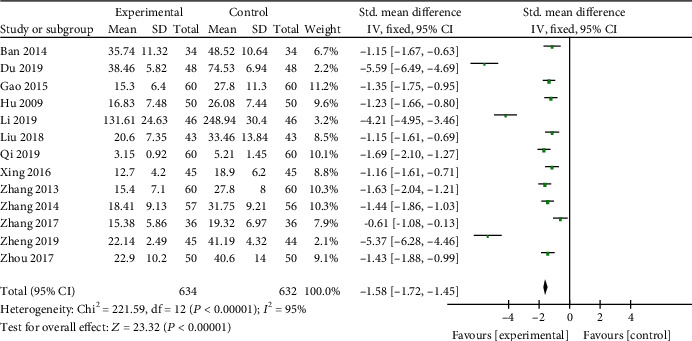
The forest plot: effects of AM for decreasing CK-MB compared with the control group (clinical studies).

**Figure 3:**
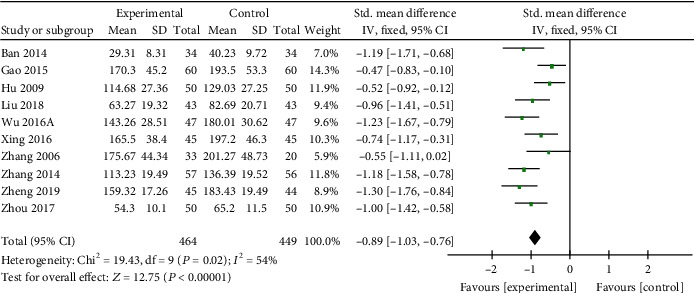
The forest plot: effects of AM for decreasing LDH compared with the control group (clinical studies).

**Figure 4:**
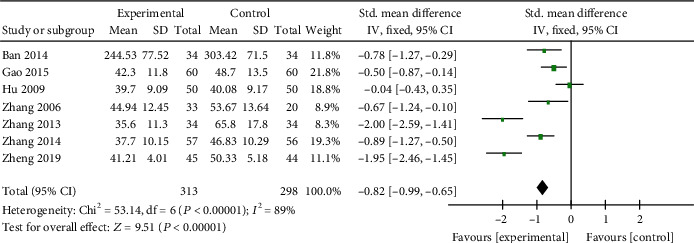
The forest plot: effects of AM for decreasing AST compared with the control group (clinical studies).

**Figure 5:**
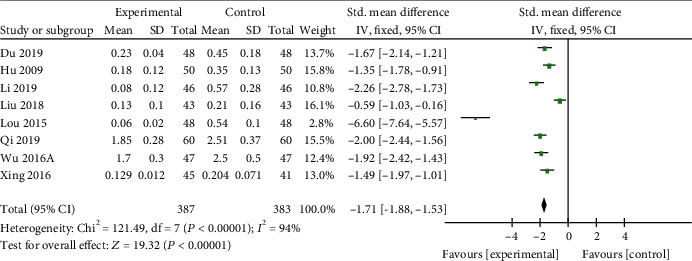
The forest plot: effects of AM for decreasing cTnI compared with the control group (clinical studies).

*(1) Cardiac Enzymes and Cardiac Troponin*. CK-MB was reported in 14 studies [19, 21, 22, 25–31, 35, 37, 38, 41], LDH in 10 studies [20, 22, 23, 25, 27, 30, 35, 38, 41, 45], AST in 7 studies [19, 25, 27, 38, 41, 42, 45], and cTnI in 8 studies [21, 23, 26, 28–30, 35, 37] as primary outcome measures. Among the studies involve in CK-MB, 1 study [37] was designed to contrast the efficacy of AI and placebo for VM; 13 studies [19, 21, 22, 25–31, 35, 38, 41] were designed to contrast the efficacy of AI plus basic treatment and basic treatment. Meta-analysis of the 13 studies revealed significant effects of AI plus basic treatment on decreasing CK-MB in patients with VM (*n* = 1266, SMD −1.58, 95% CI [−1.72 to −1.45], *P* < 0.00001; heterogeneity: *χ*^2^ = 221.59, df = 12 (*P* < 0.00001); *I*^2^ = 95%, Figure 2). The remaining one study [37] showed that CK-MB was decreased evidently by AI contrast with the placebo group. Meta-analysis of 10 studies and 7 studies showed separately that AI plus basic treatment could decrease LDH (*n* = 913, SMD −0.89, 95% CI [−1.03 to −0.76], *P* < 0.00001; heterogeneity: *χ*^2^ = 19.43, df = 9 (*P* = 0.02); *I*^2^ = 54%, Figure 3) and AST (*n* = 611, SMD −0.82, 95% CI [−0.99 to −0.65], *P* < 0.00001; heterogeneity: *χ*^2^ = 53.14, df = 6 (*P* < 0.00001); *I*^2^ = 89%, Figure 4) significantly in patients with VM. As for cTnI, meta-analysis of 8 studies showed significant effects of AI plus basic treatment on reducing cTnI in patients with VM (*n* = 770, SMD −1.71, 95% CI [−1.88 to −1.53], *P* < 0.00001; heterogeneity: *χ*^2^ = 121.49, df = 7 (*P* < 0.00001); *I*^2^ = 94%, Figure 5). The heterogeneity did not decrease significantly after sensitive analysis or removing any study involve in CK-MB, AST, or cTnI.

**Figure 6:**
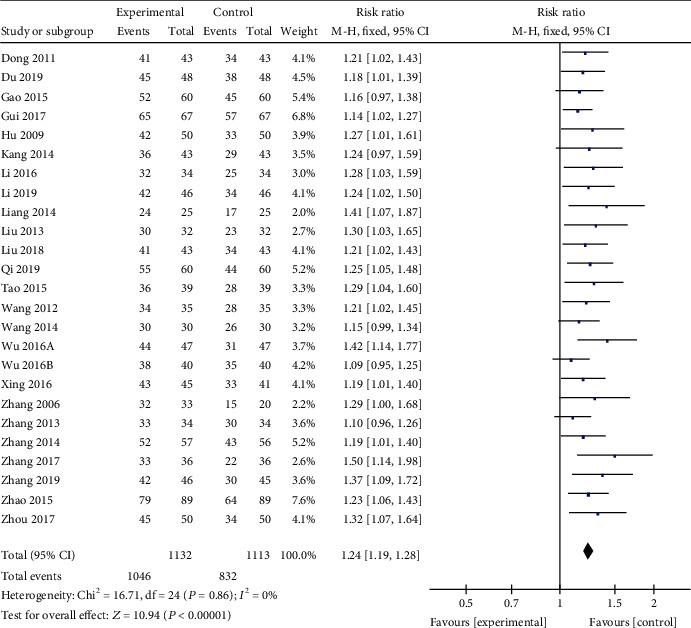
The forest plot: effects of AM for increasing the effective rate of clinical treatment compared with the control group.

**Figure 7:**
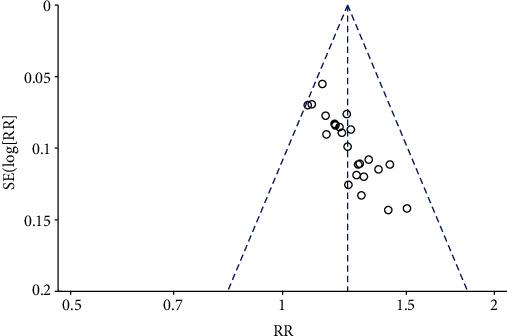
The funnel plot: effects of AM on an effective rate of clinical treatment.

*(2) Effective Rate of Clinical Treatment*. The effective rate of clinical treatment was reported in 25 studies [19–23, 25–28, 30–36, 38–46] to contrast the efficacy of AI or AM granule plus basic treatment and basic treatment, except 1 comparative study [37] of AI and placebo. Meta-analysis of the 25 studies showed significant effects of AI plus basic treatment on increasing the effective rate of clinical treatment compared with basic treatment (*n* = 2245, RR 1.24, 95% CI [1.19 to 1.28], *P* < 0.00001; heterogeneity: *χ*^2^ = 16.71, df = 24 (*P* = 0.86); *I*^2^ = 0%, Figure 6). The symmetrical publication bias funnel indicated that there is no obvious publication bias in this study (Figure 7). The remaining 1 study also showed that the efficacy of AI in the treatment of VM was significantly better than that in the placebo group (*P* < 0.05).

*(3) Adverse Reactions*. Adverse reactions were reported in 10 studies [19, 26–29, 32, 33, 36, 37, 39]. Serious adverse reactions such as liver and kidney function injury, anaphylactic shock, carcinogenesis, and teratogenesis were not mentioned in the included studies. No statistical difference was found in gastrointestinal discomfort reported as the most common adverse reaction (*P* > 0.05).

#### 3.4.2. Outcomes of Animal Studies

**Figure 8:**
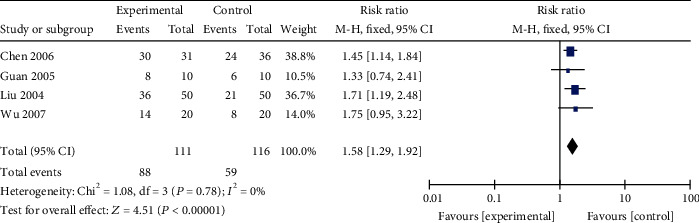
The forest plot: effects of AM for increasing the survive rate of VM animals.

*(1) Survive Rate*. A meta-analysis of 4 studies [13, 54, 57, 58] showed that AM induces a significant improvement in the survive rate of VM animals, compared with the control group (*n* = 227, RR 1.58, 95% CI [1.29 to 1.92], *P* < 0.0001; heterogeneity: *χ*^2^ = 1.08, df = 3 (*P* = 0.78); *I*^2^ = 0%, Figure 8).

**Figure 9:**
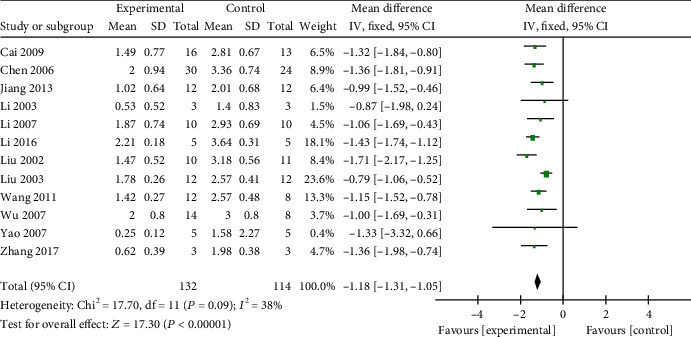
The forest plot: effects of AM for decreasing cardiac pathological score compared with the control group (animal studies).

*(2) Cardiac Pathology*. Cardiac pathology was reported in 15 studies [13, 47–55, 57–61]. Among them, 12 studies [13, 47–51, 53–55, 59–61] calculated cardiac pathological score with reference to the method proposed by Siasos et al. [62]. Meta-analysis of these studies showed significant effects of AM on reducing cardiac pathological score in animals with VM (*n* = 246, MD −1.18, 95% CI [−1.31 to −1.05], *P* < 0.0001; heterogeneity: *χ*^2^ = 17.70, df = 11 (*P* = 0.09); *I*^2^ = 38%, Figure 9). Among other studies, AM treatment significantly promoted the growth of cardiac fibroblasts in 1 study [52]. Astragalus inhibited the hypertrophy of cardiomyocyte in 1 study [58]. Astragalus inhibited the infiltration of inflammatory cell in 1 study [57].

**Figure 10:**
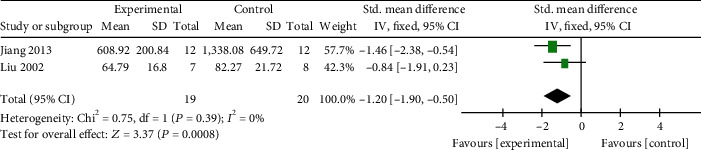
The forest plot: effects of AM for decreasing CK-MB compared with the control group (animal studies).

**Figure 11:**
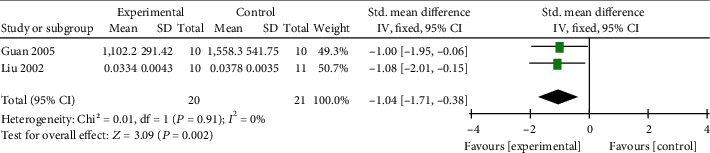
The forest plot: effects of AM for decreasing LDH compared with the control group (animal studies).

**Figure 12:**
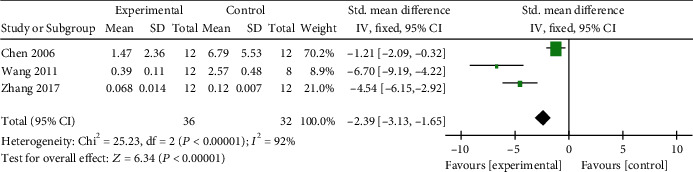
The forest plot: effects of AM for decreasing cTnI compared with the control group (animal studies).

*(3) Cardiac Enzymes and Cardiac Troponin*. Meta-analysis of 3 studies [47, 49, 61] indicated significant effects of AM on reducing CK-MB in VM animals compared with control group (*n* = 63, SMD −1.65, 95% CI [−2.33 to −0.98], *P* < 0.00001; heterogeneity: *χ*^2^ = 25.62, df = 2 (*P* < 0.00001); *I*^2^ = 92%). After sensitivity analyses, we removed 1 study [47] that used AM at a dose of 0.4 ml/g. Meta-analysis of 2 studies [49, 61] showed significant effects of AM on reducing CK-MB (*n* = 39, SMD −1.20, 95% CI [−1.90 to −0.50], *P* < 0.00001; heterogeneity: *χ*^2^ = 0.75, df = 1 (*P* = 0.39); *I*^2^ = 0%, Figure 10). Meta-analysis of 2 studies [57, 61] showed significant effects of AM on decreasing LDH compared with the control group (*n* = 42, SMD −1.04, 95% CI [−1.71 to −0.38], *P* < 0.00001; heterogeneity: *χ*^2^ = 0.01, df = 1 (*P* = 0.91); *I*^2^ = 0%, Figure 11). Meta-analysis of 3 studies [13, 47, 50] showed significant effects of AM on decreasing cTnI compared with the control group (*n* = 68, SMD −2.39, 95% CI [−3.13 to −1.65], *P* < 0.00001; heterogeneity: *χ*^2^ = 25.23, df = 2 (*P* < 0.00001); *I*^2^ = 92%, Figure 12). The heterogeneity did not decrease significantly after sensitive analysis or removing any study involve in cTnI.

**Figure 13:**
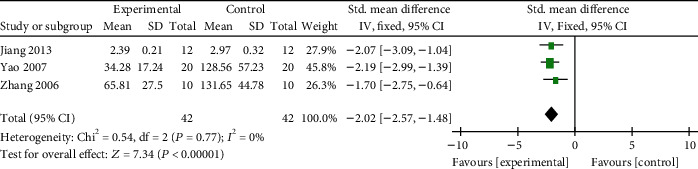
The forest plot: effects of AM for decreasing TNF-*α* compared with the control group (animal studies).

*(4) Cardioprotective Mechanisms*. Meta-analysis of 3 studies [49, 53, 56] showed significant effects of AM on decreasing TNF-*α* compared with the control group in VM animal (*n* = 84, SMD −2.02, 95% CI [−2.57 to −1.48], *P* < 0.00001; heterogeneity: *χ*^2^ = 0.54, df = 2 (*P* = 0.77); *I*^2^ = 0%, Figure 13); 1 study [52] for reducing IL-2, IL-8, and IL-18 (*P* < 0.05); 1 study [51] for reducing MCP-1 (*P* < 0.05); 1 study [50] for reducing MIP-2 (*P* < 0.05); 1 study [57] for reducing MDA (*P* < 0.05) and increasing SOD (*P* < 0.05); 1 study [55] for reducing the expression of CAR (*P* < 0.05); 1 study [48] for reducing Cav-3 and Smad3 (*P* < 0.05); 1 study [13] for reducing ET-1, ANP, and ETR Bmax (*P* < 0.05) and increasing the activity of SERCA (*P* < 0.05), and 1 study [60] for reducing the expression of Fas and FasL (*P* < 0.05).

### 3.5. Subgroup Analysis

**Figure 14:**
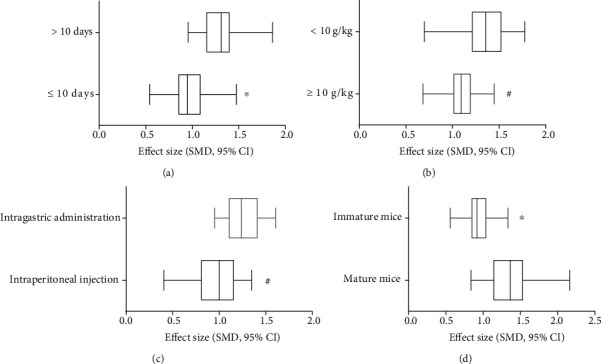
Effect of AM on cardiac pathological score in subgroups. (a) Duration of treatment; (b) AM dose; (c) induction type; (d) age of animals. ^#^*P* > 0.05 vs. control groups; ^∗^*P* ≤ 0.05 vs. control groups.

The potential confounding factors (including age of animals, varying methods of administration, varying doses of AM, and various durations of treatment) that may increase the heterogeneity of outcome measures were explored using stratified analysis of cardiac pathological score. In the subgroup analysis of age of Balb/c mice, the effect size of the model used mature mice (≥6 weeks) showed better results than immature mice (<6 weeks) (SMD −1.40 vs. SMD −0.97, *P* = 0.009, Figure 14(d)), and the heterogeneity of two groups decreased obviously. No difference was seen between the intraperitoneal injection group and oral gavage group (SMD −1.01 vs. SMD −1.28, *P* = 0.06, Figure 14(c)). The heterogeneity of the two groups decreased insignificantly. In the subgroup analysis of durations of treatment, the longer period of AM treatment (>10 days) showed better effect size than the shorter treatment (≤10 days) (SMD −1.28 vs. SMD −1.01, *P* = 0.05, Figure 14(a)), and the heterogeneity of the longer period group decreased significantly. No difference was seen between the high dose of AM group (≥10 g/kg) and low-dose group (<10 g/kg) (SMD −1.08 vs. SMD −1.15, *P* = 0.15, Figure 14(b)), and the heterogeneity of two groups decreased insignificantly.

## 4. Discussion

### 4.1. Summary of Evidence

This is a first-ever systematic review, which includes 28 randomized clinical control studies with 2522 participants and 16 animal studies with 634 animals to comprehensively and systematically evaluate the efficacy, safety, and possible mechanisms of AM in the treatment of VM. The quality of the studies included was generally moderate. The evidence available from the present study showed a cardioprotective function of AM for VM animals and patients by multiple mechanisms.

### 4.2. Limitations

There are some limitations of the present study: (1) English and Chinese literatures were included only in the present study, which may lead to a certain degree of selection bias; (2) All patients were patients with mild viral myocarditis, which may exaggerate the therapeutic effect of AM; (3) clinical adverse reactions were seldom to be reported; (4) most of the included clinical studies are short-term follow-up studies with small sample size; (5) the studies selected for our analysis had methodological deficiencies, such as seldom using allocation concealment and the blind method.

### 4.3. Implications

The results of subgroup analysis showed that AM reduced the cardiac pathological score of mature Balb/c mice with VM significantly better than that of immature Balb/c mice (SMD −1.40 vs. SMD −0.97, *P* = 0.009), which suggests that the age of mice may be the source of high heterogeneity. It may be related to CAR which is the receptor that binds to the Coxsackie virus on cardiomyocytes [63]. The study from Li and Yi showed that the expression of CAR in the myocardium of mice infected with CVB3 increased significantly and reached a peak on the 7th day after infection, and the disease was aggravated simultaneously [55]. However, the expression of CAR decreased significantly after AM treatment [55]. Thus, we draw a conclusion that CAR plays a key role in the process of infection of CVB3 into target cells, and AM was able to downregulate it. Ito et al. [64] found that CAR was abundant in the hearts of newborn rats but was barely detectable in the hearts of adult rats, which is regarded as one of the crucial reasons that CVB3 tends to infect children and causes severe impact. In addition, eliminating CAR was found to prevent signs of inflammatory cardiomyopathy, with essentially no pathology in animal hearts [65]. And the deletion of CAR at the later stage of mice embryo (≥11days) has no effect on the survival of many embryos to adulthood and heart development [66]. Thus, the development of drugs that inhibit the expression of CAR may be an important direction in the future treatment of VM, especially in children.

The results of another subgroup analysis showed that the longer period of AM treatment (>10 days) showed better effect size than the shorter treatment (≤10 days) (SMD −1.28 vs. SMD −1.01, *P* = 0.05), which suggests that the duration of treatment may be the source of high heterogeneity. Myocardial injury caused by VM can be subdivided into two stages. In the early few days of the VM, virus replication causes the exposure of intracellular antigens, myocyte necrosis, and activation of the host's immune system. The specific performance is the invasion of NK cells and macrophages followed by T lymphocytes. The subacute stage covers few weeks to several months [7]. It is characterized by activated virus-specific T lymphocytes, which may target the host's organs by molecular mimicry. Two studies [55, 59] reported that AM inhibited the replication of CVB3 and directly reduced the cardiac damage caused by viral replication at the acute stage. In addition, AM also inhibited the activation of T lymphocytes by inhibiting the expression of cytokines (TNF-*α* [49, 53, 56], IL-8 [52], MCP-1 [51], and MIP-2 [50]) and reducing myocardial injury at the immune reactions stage (subacute stage). The evidences above suggest that long-term (≥10 days) AM treatment may bring greater benefits to VM. However, there are few studies on multiple time points to measure the main outcome indicators at the current stage. Thus, we suggest that further clinical studies or animal experiments could verify the above theory.

The therapeutic effect of myocarditis was significantly related to the severity of the disease. However, in all the animal studies included, no classification of the mice according to the severity of myocarditis was done. Meanwhile, in all the clinical trials, patients were all with mild viral myocarditis, and no deaths were reported. Thus, with the available primary data, it is impossible to do subgroup analysis according to disease severity. We recommend that the severity of myocarditis should be considered and classified in future studies.

It is reported that low-quality trials have a statistically significant 30–50% exaggeration of treatment efficacy compared with high-quality trials [67]. The quality of the included studies in the present study was considered to be moderate to inferior, with 3-7 points for clinical studies, and 2-5 points for animal studies. Most of the studies had methodological deficiencies, such as seldom using allocation concealment and the blind method. In addition, except for the major projects supported by the fund, few studies have registered experiments in advance or published protocols, which may lead to selective reporting bias [68]. Poor experimental design is a major obstacle to translating preclinical animal research into clinical treatments for human diseases [68]. Thus, we recommend that clinical research should refer to the CONSORT (Consolidated Standards of Reporting Trials) 2010 statement [69], animals research should refer to the ARRIVE (The Animal Research: Reporting In Vivo Experiments) guidelines [70], and the use of allocation concealment and blinding should pay more attention to both clinical and animal research. Moreover, multiple details related to animal treatment, such as anesthesia, analgesia, nutrition, environment (temperature, humidity), and euthanasia, should be recorded in detail, as the lack of humane treatment for animals may also affect the accuracy of the results [70]. Animal research should be registered prior to its execution in a generally accessible database (http://www.crd.york.ac.uk/PROSPERO), and clinical research should be registered (http://www.clinicaltrials.com). It allows verification of the predefined study hypothesis and end-points of the study and reduces publication bias [71].

**Figure 15:**
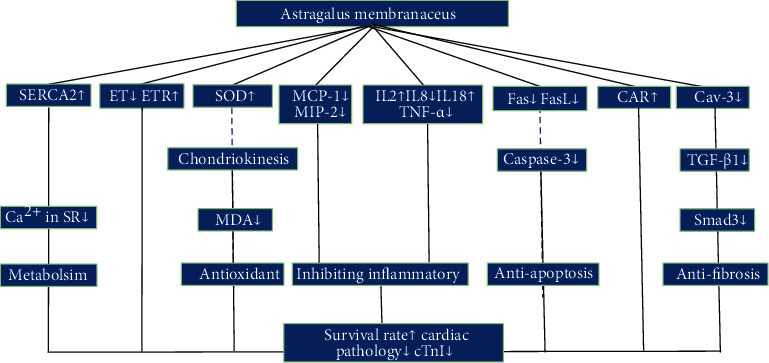
A schematic representation of mechanisms of AM for VM.

The possible mechanisms of AM mediated cardioprotection in the included studies are summed up as follows: (1) anti-inflammation by reducing TNF-*α* [49, 53, 56], IL-8 [52], MCP-1 [51], and MIP-2 [50] and increasing IL-18 and IL-2 [52]; (2) antioxidant effects by increasing SOD to reduce the release of MDA [57]; (3) alleviating myocardial fibrosis by inhibiting Cav-3 and TGF-*β*1 to reduce the expression of Smad3 [48]; (4) inhibiting apoptosis by downregulating gene transcription of Fas/Fasl and reducing the expression of caspase-3 [59]; (5) reducing the calcium overload in sarcoplasmic reticulum to maintain diastolic and systolic of cardiomyocytes by enhancing the activity of SERCA [13]; (6) improving cardiac remodeling by upregulating ETR affinity and reducing the expression of ET-1 and ANP [13]; and (7) inhibiting virus infection and replication by reducing the expression of CAR [55]. The mechanism is summarized in Figure 15.

## 5. Conclusion

Our findings indicate that AM exerted cardioprotective function in VM animals and patients largely through antivirus and antivirus receptors, anti-inflammatory, antioxidation, antiapoptotic, antifibrosis, and reducing cardiac calcium load. However, due to methodological deficiencies in the original study, current research results need to be treated with caution, and further evidence from future high-quality clinical and animal studies is needed. In conclusion, AM is a potential cardioprotective candidate in the treatment of VM.

## Figures and Tables

**Table 1:** The characteristic of clinical studies.

Study (years)	Number (*n* = male/female); mean age (years); course of disease (days)		Treatments		Duration of treatment	Adverse reactions	Outcome index	Intergroup differences
	Experimental group	Control group	Experimental group	Control group				
Du 2019	48 (25/23)	48 (26/22)	By intravenous drip infusion of AM injection (20 ml, qd) in 5% GS 250 ml + basic treatment	Antiviral drugs + nutritional myocardial drugs + sodium creatine phosphate for injection (1 g, qd)	2 weeks	Y	(1) Effective rate (2) CK (3) CK-MB (4) cTnI	(1) *P* < 0.05 (2) *P* < 0.05 (3) *P* < 0.05 (4) *P* < 0.05
	7.81 ± 2.24	7.63 ± 2.16						
	NM	NM						

Li et al. 2019	46 (25/21)	46 (26/20)	By intravenous drip infusion of AM injection (2 ml/kg, qd) in NS 250 ml + basic treatment	Antiviral drugs + nutritional myocardial drugs	2 weeks	Y	(1) Effective rate (2) CK (3) CK-MB (4) cTnI (5) TNF-*α* (6) IL-6 (7) Effect of arrhythmia	(1) *P* < 0.05 (2) *P* < 0.05 (3) *P* < 0.05 (4) *P* < 0.05 (5) *P* < 0.05 (6) *P* < 0.05 (7) *P* < 0.05
	5.67 ± 1.82	5.83 ± 1.72						
	NM	NM						

Qi et al. 2019	60 (NM)	60 (NM)	By intravenous drip infusion of AM injection (20 ml, qd) in 5% GS 500 ml + basic treatment	Adenosine disodium triphosphate, coenzyme A and insulin for injection (20 mg, qd) + fructose sodium diphosphate injection (5 g, bid)	4 weeks	N	(1) Effective rate (2) hs-CRP (3) CK-MB (4) cTnI (5) TNF-*β* (6) IL-10 (7) IL-17 (8) IL-21 (9) miR-146b (10) miR-155	(1) *P* < 0.05 (2) *P* < 0.05 (3) *P* < 0.05 (4) *P* < 0.05 (5) *P* < 0.05 (6) *P* < 0.05 (7) *P* < 0.05 (8) *P* < 0.05 (9) *P* < 0.05 (10) *P* < 0.05
	NM	NM						
	NM	NM						

Zhang et al. 2019	46 (22/24)	45 (20/25)	By intravenous drip infusion of AM injection (20 ml, qd) in 5% GS 200 ml + basic treatment	Antiviral drugs (ribavirin) + vitamin C + Inosine injection + fructose 1,6-diphosphate injection	2 weeks	N	(1) Effective rate (2) CD3^+^ (3) CD4^+^ (4) CD8^+^ (5) CD4^+^/CD8^+^ (6) EF	(1) *P* < 0.05 (2) *P* < 0.05 (3) *P* < 0.05 (4) *P* < 0.05 (5) *P* < 0.05 (6) *P* < 0.05
	36.12 ± 5.87	35.52 ± 5.47						
	NM	NM						

Zheng 2019	45 (21/24)	44 (23/21)	By intravenous drip infusion of AM injection (20 ml, qd) in NS 250 ml + basic treatment	Antiviral drugs + nutritional myocardial drugs + trimetazidine	6 weeks	Y	(1) Recover time of clinical symptoms (2) Recovery time of arrhythmia (3) CK-MB (4) AST (5) LDH	(1) *P* < 0.05 (2) *P* < 0.05 (3) *P* < 0.05 (4) *P* < 0.05 (5) *P* < 0.05
	48.47 ± 15.25	49.15 ± 16.18						
	15.11 ± 3.25	16.01 ± 3.31						

Liu et al. 2018	43 (20/23)	43 (24/19)	By intravenous drip infusion of AM injection (0.5 ml/kg, qd) in 5% GS 250 ml + basic treatment	Antiviral drugs + vitamin C + fructose + coenzyme Q10 + creatine phosphate injection (1.0 g, qd)	2 weeks	N	(1) Effective rate (2) CK (3) CK-MB (4) LDH (5) cTnI	(1) *P* < 0.05 (2) *P* < 0.05 (3) *P* < 0.05 (4) *P* < 0.05 (5) *P* < 0.05
	7.36 ± 1.48	7.74 ± 1.65						
	NM	NM						

Gui 2017	67 (34/33)	67 (35/32)	By oral administration of AM granule (age ≤ 2 years 1 g, bid; 2 < age ≤ 4 years, 1.5 g, bid; 4 < age ≤ 6 years, 2 g, bid; age > 6 years, 4 g, bid) + basic treatment	Vitamin C + energy mixture injection + vitamin E + coenzyme Q + coenzyme A	4 weeks	Y	(1) Effective rate	(1) *P* < 0.05
	5.4 ± 0.5	5.7 ± 0.3						
	NM	NM						

Zhang et al. 2017B	36 (20/16)	36 (14/22)	By intravenous drip infusion of AM injection (10 ml, qd) in 5% GS + basic treatment	Antiviral drugs (recombinant human interferon *α*2b injection) + vitamin C + coenzyme Q10	4 weeks	N	(1).Effective rate (2) CK (3) CK-MB (4) TNF-*α* (5) IL-10 (6) IL-6	(1) *P* < 0.05 (2) *P* < 0.05 (3) *P* < 0.05 (4) *P* < 0.05 (5) *P* < 0.05 (6) *P* < 0.05
	5.29 ± 2.91	5.38 ± 2.86						
	NM	NM						

Zhou 2017	50 (26/24)	50 (28/22)	By intravenous drip infusion of AM injection (5-10 ml, qd) in 5% GS 150 ml + basic treatment	Antiviral drugs (ribavirin) + vitamin C + coenzyme Q10	15 days	N	(1) Effective rate (2) CK (3) CK-MB (4)LDH	(1) *P* < 0.05 (2) *P* < 0.05 (3) *P* < 0.05 (4) *P* < 0.05
	35.9 ± 2.7	36.4 ± 3.4						
	NM	NM						

Li et al. 2016A	34 (21/13)	34 (19/15)	By intravenous drip infusion of AM injection (5-10 ml, qd) in 5% GS 150 ml + basic treatment	Vitamin C + vitamin E + coenzyme Q10 + energy mixture injection	2 weeks	N	(1) Effective rate (2) INF-*γ* (3) IL-4 (4) INF-*γ*/IL-4	(1) *P* < 0.05 (2) *P* < 0.05 (3) *P* < 0.05 (4) *P* < 0.05
	8.54 ± 4.38	9.14 ± 5.26						
	6.21 ± 3.17	5.26 ± 2.79						

Xing 2016	45 (29/16) 5.4 ± 1.7 14.7 ± 3.2	41 (28/13) 5.1 ± 1.6 15.1 ± 3.4	By intravenous drip infusion of AM injection (5-10 ml, qd) in 5% GS 100 ml + basic treatment	Antiviral drugs + vitamin C + coenzyme Q10	3 weeks	N	(1) Effective rate (2) CK (3) CK-MB (4) LDH (5) cTnI (6) CD3^+^ (7) CD4^+^ (8) CD8^+^	(1) *P* < 0.01 (2) *P* < 0.01 (3) *P* < 0.01 (4) *P* < 0.01 (5) *P* < 0.01 (6) *P* < 0.01 (7) *P* < 0.05 (8) *P* < 0.05

Wu 2016	47 (21/26)	47 (24/23)	By intravenous drip infusion of AM injection (20 ml, qd) in 5% GS 250 ml + basic treatment	Antiviral drugs (recombinant human interferon *α*2b injection) + vitamin C + energy mixture injection	2 weeks	N	(1) Effective rate (2) LDH (3) cTnI (4) CD3^+^ (5) CD4^+^ (6) CD8^+^ (7) NK	(1) *P* < 0.05 (2) *P* < 0.01 (3) *P* < 0.01 (4) *P* < 0.01 (5) *P* < 0.01 (6) *P* < 0.01 (7) *P* < 0.01
	26.7 ± 10.1	26.4 ± 10.5						
	NM	NM						

Wu et al. 2016	40 (24/16)	40 (22/18)	By intravenous drip infusion of AM injection (10-20 ml, qd) in NS 50 ml + basic treatment	Antiviral drugs + fructose	2 weeks	Y	(1) Effective rate (2) CD3^+^ (3) CD4^+^ (4) CD8^+^ (5) NK	(1) *P* < 0.05 (2) *P* < 0.05 (3) *P* < 0.05 (4) *P* < 0.05 (5) *P* < 0.05
	6.48 ± 1.40	6.44 ± 1.43						
	2.13 ± 0.68	2.11 ± 0.66						

Gao 2015	60 (27/33)	60 (25/35)	By intravenous drip infusion of AM injection (20 g, qd) in 5% GS 500 ml + basic treatment	Polarization liquid + vitamin C + coenzyme Q10	2 weeks	N	(1) Effective rate (2) CK (3) CK-MB (4) LDH (5) AST	(1) *P* < 0.05 (2) *P* < 0.05 (3) *P* < 0.05 (4) *P* < 0.05 (5) *P* < 0.05
	38.5 ± 7.2	37.4 ± 7.0						
	31.2 ± 6.9	32.1 ± 8.0						

Lou 2015	48 (34/14)	48 (25/23)	By intravenous drip infusion of AM injection (20-30 ml, qd) in 5% GS 250 ml	Polarization liquid	2 weeks	Y	(1) Effective rate (2) TNF-*α* (3) INF-8 (4) INF-6 (5) CK (6) CK-MB (7) cTnI	(1) *P* < 0.05 (2) *P* < 0.05 (3) *P* < 0.05 (4) *P* < 0.05 (5) *P* < 0.05 (6) *P* < 0.05 (7) *P* < 0.05
	6.01 ± 1.2	5.8 ± 1.5						
	NM	NM						

Tao 2015	39 (22/17)	39 (24/15)	By intravenous drip infusion of AM injection (1 ml/kg, qd) in 5% GS 100 ml + basic treatment	Antiviral drugs (ribavirin) + coenzyme A + ATP + vitamin C	15 days	Y	(1) Effective rate (2) Recover time of clinical symptoms (3) Recover time of clinic syndrome myocardial enzyme	(1) *P* < 0.05 (2) *P* < 0.05 (3) *P* < 0.05
	6.2 ± 2.4	6.6 ± 2.7						
	7.5 ± 7.6	7.2 ± 7.3						

Zhao et al. 2015	89 (51/38)	89 (44/45)	By intravenous drip infusion of AM injection (20-30 ml, qd) in 5% GS 250 ml + basic treatment	Antiviral drugs (ribavirin) + vitamin C + coenzyme Q10 + inosine tablets + polarization liquid	2 weeks	N	(1) Effective rate (2) SOD (3) MDA (4) GST (5) NO (6) TNF-*α* (7) INF-*γ* (8) IL-8 (9) IL-6	(1) *P* < 0.05 (2) *P* < 0.05 (3) *P* < 0.05 (4) *P* < 0.05 (5) *P* < 0.05 (6) *P* < 0.05 (7) *P* < 0.05 (8) *P* < 0.05 (9) *P* < 0.05
	29.4 ± 11.8	30.1 ± 12.7						
	NM	NM						

Ban 2014	34 (20/14)	34 (18/16)	By intravenous drip infusion of AM injection (2 g/kg, qd) in 5% GS 250 ml + basic treatment	Coenzyme Q10 + ATP + vitamin C	4 weeks	N	(1) CK (2) CK-MB (3) LDH (4) AST (5) *α*-HBDH	(1) *P* < 0.05 (2) *P* < 0.05 (3) *P* < 0.05 (4) *P* < 0.05 (5) *P* < 0.05
	8 ± 2.01	9 ± 1.28						
	NM	NM						

Wang et al. 2014	30 (16/14)	30 (18/12)	By intravenous drip infusion of AM injection (20 ml, qd) in 10% GS 250 ml + basic treatment	Antiviral drugs (acyclovir) + vitamin C + coenzyme A	46 days	Y	(1) Effective rate	(1) *P* < 0.05
	9.3 ± 2.8	12.1 ± 2.2						
	NM	NM						

Liang 2014	25 (14/11)	25 (13/12)	By intravenous drip infusion of AM injection (10-20 ml, qd) in 5% GS 100 ml + basic treatment	Antiviral drugs + vitamin C + coenzyme Q10	4 weeks	N	(1) Effective rate (2) SOD (3) MDA (4) NO	(1) *P* < 0.05 (2) *P* < 0.05 (3) *P* < 0.05 (4) *P* < 0.05
	7.9 ± 3.6	8.1 ± 3.2						
	16.2 ± 2.5	14.1 ± 2.3						

Kang 2014	43 (22/21)	43 (21/22)	By intravenous drip infusion of AM injection (30 ml, qd) in 10% GS 250 ml	NM	15 days	N	(1) Effective rate (2) Effect of arrhythmia	(1) *P* < 0.05 (2) *P* < 0.05
	NM	NM						
	NM	NM						

Zhang 2014	57 (32/25)	56 (32/24)	By oral administration of AM granule (age ≤ 2 years, 1 g, bid; 2 < age ≤ 4 years, 1.5 g, bid; 4 < age ≤ 6 years, 2 g, bid; age > 6 years, 4 g, bid) + basic treatment	Antiviral drugs (ribavirin) + fructose + coenzyme Q10 + vitamin C + gamma globulin	2 weeks	N	(1) Effective rate (2) CK-MB (3) LDH (4) AST	(1) *P* < 0.05 (2) *P* < 0.05 (3) *P* < 0.05 (4) *P* < 0.05
	3.7 ± 1.5	3.8 ± 1.5						
	7.4 ± 0.8	7.3 ± 0.8						

Liu et al. 2013	32 (18/14)	32 (20/12)	By intravenous drip infusion of AM injection (5-10 ml, qd) in 5% GS 100 ml + basic treatment	NM	2 weeks	N	(1) Effective rate (2) IL-23 (3) IL-17 (4) Th17 cell	(1) *P* < 0.05 (2) *P* < 0.05 (3) *P* < 0.05 (4) *P* < 0.05
	9.1 ± 5.1	8.7 ± 4.5						
	6.2 ± 3.1	5.2 ± 2.8						

Zhang et al. 2013	34 (17/17)	34 (17/17)	By intravenous drip infusion of AM injection (50 ml, qd) in 5% GS 250 ml + basic treatment	Energy mixture injection + vitamin C + coenzyme Q10 + polarization liquid	2 weeks	N	(1) Effective rate (2) CK-MB (3) ESR (4) AST (5) ALT	(1) *P* < 0.05 (2) *P* < 0.05 (3) *P* < 0.05 (4) *P* < 0.05 (5) *P* < 0.05
	NM	NM						
	NM	NM						

Wang 2012	35 (18/17)	35 (20/15)	By intravenous drip infusion of AM injection (50 ml, qd) + basic treatment	Antiviral drugs + vitamin C + coenzyme Q10 + polarization liquid + Trimetazidine	4 weeks	Y	(1) Effective rate (2) Arrhythmia (3) cTnI (4) Cardiac function	(1) *P* < 0.05 (2) *P* < 0.05 (3) *P* < 0.05 (4) *P* < 0.05
	55 ± 11	60 ± 9						
	53 ± 17	55 ± 16						

Dong 2011	60 (34/26)	50 (28/22)	By intravenous drip infusion of AM injection (5-20 ml, qd) + basic treatment	Antiviral drugs (ribavirin) + vitamin C + fructose + energy mixture injection	30 days	N	(1) Effective rate (2) Recovery rate of CK-MB (3) Recovery rate of CK (4) Recovery rate of LDH (5) Recovery rate of AST	(1) *P* < 0.05 (2) *P* < 0.05 (3) *P* < 0.05 (4) *P* < 0.05 (5) *P* < 0.05
	6.5	6.9						
	NM	NM						

Hu 2009	50 (26/24)	50 (27/23)	By intravenous drip infusion of AM injection (60 ml, bid) in 5% GS 250 ml + basic treatment	Coenzyme Q10 + polarization liquid	2 weeks	Y	(1) Effective rate (2) Effect of arrhythmia (3) AST (4) CK-MB (5) LDH (6) cTnI (7) NK (8) TNF-*α* (9) IL-1 (10) IL-6	(1) *P* < 0.05 (2) *P* < 0.05 (3) *P* > 0.05 (4) *P* < 0.05 (5) *P* < 0.05 (6) *P* < 0.05 (7) *P* < 0.05 (8) *P* > 0.05 (9) *P* < 0.05 (10) *P* > 0.05
	38 ± 10.1	35 ± 9.8						
	4 ± 0.7 months	5 ± 0.6 months						

Zhang et al. 2006B	33 (20/13)	20 (14/6)	By intravenous drip infusion of AM injection (5-10 ml, qd) in 5% GS 150 ml + basic treatment	Antiviral drugs (ribavirin) + vitamin C + vitamin E + energy mixture injection	2 weeks	N	(1) Effective rate (2) CK (3) AST (4) LDH	(1) *P* < 0.01 (2) *P* < 0.05 (3) *P* < 0.05 (4) *P* < 0.05
	2 ± 11	2 ± 11						
	NM	NM						

Note: *α*-HBDH: *α*-hydroxybutyric dehydrogenase; hs-CRP: hypersensitive C-reactive protein; miR: microRNA; ALT: alanine aminotransferase; AM: Astragalus membranaceus; AST: aspartate aminotransferase; ATP: adenosine triphosphate; Bid: bis in die; cTnI: cardiac troponin I; CD: cluster of differentiation; CK: creatine kinase; CK-MB: creatine kinase isoenzyme; EF: ejection fraction; ESR: erythrocyte sedimentation rate; GS: glucose injection; GST: glutathione transferase; IL: interleukin; INF-*γ*: interferon-*γ*; LDH: lactic dehydrogenase; MDA: malondialdehyde; N: no; NK: natural killer cell; NM: not mentioned; NO: nitric oxide; NS: normal saline; Qd: quaque die; SOD: superoxide dismutase; Th: T helper cell; TNF: tumor necrosis factor; Y: yes.

**Table 2:** Information of AM of clinical study.

Study (years)	Specifications	Source	Concentration (crude drug content)	Quality control reported
Du 2019	Injection	Heilongjiang Zhenbaodao Pharmaceutical Co., Ltd.	2 g/mL	Traditional Chinese patented medicine WY: Z23020782
Li et al. 2019	Injection	Jiangsu Jiuxu Pharmaceutical Co., Ltd.	2 g/mL	Traditional Chinese patented medicine WY: Z19993151
Qi et al. 2019	Injection	Heilongjiang Zhenbaodao Pharmaceutical Co., Ltd.	2 g/mL	Traditional Chinese patented medicine WY: Z23020782
Zhang et al. 2019	Injection	Jiangsu Jiuxu Pharmaceutical Co., Ltd.	2 g/mL	Traditional Chinese patented medicine WY: Z20003189
Zheng 2019	Injection	Shenwei Pharmaceutical Group Co., Ltd.	2 g/mL	Traditional Chinese patented medicine WY: Z13020999
Liu et al. 2018	Injection	Unknown	Unknown	Unknown
Gui 2017	Granule	Unknown	Unknown	Unknown
Zhang et al. 2017B	Injection	Unknown	Unknown	Unknown
Zhou 2017	Injection	Unknown	Unknown	Unknown
Li et al. 2016A	Injection	Chengdu Di'ao Jiuhong Pharmaceutical Factory	2 g/mL	Batch number: 0210094
Xing 2016	Injection	Shanghai Hefeng Pharmaceutical Co., Ltd.	2 g/mL	Batch number: 20120829
Wu 2016	Injection	Shenwei Pharmaceutical Group Co., Ltd.	2 g/mL	Traditional Chinese patented medicine WY: Z13020999
Wu et al. 2016	Injection	Unknown	Unknown	Unknown
Gao 2015	Injection	Unknown	Unknown	Unknown
Lou 2015	Injection	Zhengda Qingchunbao Pharmaceutical Co., Ltd.	2 g/mL	Traditional Chinese patented medicine WY: Z33020178
Tao 2015	Injection	Shenwei Pharmaceutical Group Co., Ltd.	2 g/mL	Traditional Chinese patented medicine WY: Z13020999
Zhao et al. 2015	Injection	Harbin Shengtai Biopharmaceutical Co., Ltd.	2 g/mL	Traditional Chinese patented medicine WY: Z23020820
Ban 2014	Injection	Unknown	Unknown	Unknown
Wang et al. 2014	Injection	Dali Pharmaceutical Co., Ltd.	2 g/mL	Traditional Chinese patented medicine WY: Z53021585
Liang 2014	Injection	Heilongjiang Zhenbaodao Pharmaceutical Co., Ltd.	2 g/mL	Batch number: 100226
Kang 2014	Injection	Unknown	Unknown	Unknown
Zhang 2014	Granule	Unknown	Unknown	Unknown
Liu et al. 2013	Injection	Chengdu Di'ao Jiuhong pharmaceutical factory	2 g/mL	Batch number: 0210094
Zhang et al. 2013	Injection	Unknown	Unknown	Unknown
Wang 2012	Injection	Unknown	Unknown	Unknown
Dong 2011	Injection	Zhengda Qingchunbao Pharmaceutical Co., Ltd.	2 g/mL	Batch number: 020213210901082, 0506013
Hu 2009	Injection	Shenwei Pharmaceutical Group Co., Ltd.	2 g/mL	Traditional Chinese patented medicine WY: Z13021000
Zhang et al. 2006B	Injection	Chengdu Di'ao Jiuhong Pharmaceutical Factory	2 g/mL	Batch number: 0210094

**Table 3:** The characteristic of animal studies.

Study (years)	Species (age; sex; number = experimental/control)	Weight	Model (method)	Experimental group	Control group	Outcome index	Intergroup differences
Zhang et al. 2017A	Balb/c mice (4-6 weeks; male; 12/12)	16-18 g	By intraperitoneal injection of culture medium containing 1000 PFU/ml CVB3 virus (0.4 ml)	By intraperitoneal injection of AM injection (0.4 ml/g, qd) for 14 d after establishing model	By intraperitoneal injection of normal saline (0.1 ml/g, qd) for 14 d after establishing model	(1) Changes of cardiac pathology (2) cTnI (3) CK-MB	(1) *P* < 0.05 (2) *P* < 0.01 (3) *P* < 0.01

Li et al. 2016B	SD rats (7-9 weeks; male/female; 15/15)	182 ± 36 g	By intraperitoneal injection of culture medium containing 500 TCID_50_ CVB3 virus (0.2 ml)	By intraperitoneal injection of AM injection (1.68 g/kg, qd) for 15 d after establishing model	By intraperitoneal injection of normal saline (20 ml/kg, qd) for 15 d after establishing model	(1) Changes of cardiac pathology (2) Caveolin-3 (3) Smad-3	(1) *P* < 0.05 (2) *P* < 0.01 (3) *P* < 0.05

Jiang 2013	Balb/c mice (4-6 weeks; NM; 12/12)	NM	By intraperitoneal injection of culture medium containing 2000 TCID_50_ CVB3 virus (0.2 ml)	By intraperitoneal injection of AM injection (10 g/kg, qd) for 7 d after establishing model	By intraperitoneal injection of normal saline (10 g/kg, qd) for 7 d after establishing model	(1) Changes of cardiac pathology (2) CK-MB (3) TNF-*α*	(1) *P* < 0.01 (2) *P* < 0.01 (3) *P* < 0.01

Wang et al. 2011	Balb/c mice (4 weeks; male; 15/15)	12-16 g	By intraperitoneal injection of culture medium containing 100 TCID_50_ CVB3 virus (0.1 ml)	By intraperitoneal injection of AM injection (10 g/kg, qd) for 14 d after establishing model	By intraperitoneal injection of normal saline (10 g/kg, qd) for 14 d after establishing model	(1) Changes of cardiac pathology (2) cTnI (3) MIP-2 mRNA (4) MIP-2	(1) *P* < 0.01 (2) *P* < 0.05 (3) *P* < 0.01 (4) *P* < 0.01

Cai et al. 2009	Balb/c mice (6-8 weeks; male; 20/20)	18-22 g	By intraperitoneal injection of culture medium containing 1000 TCID_50_ CVB3 virus (0.15 ml)	By intraperitoneal injection of AM injection (10 g/kg, qd) for 14 d after establishing model	By intraperitoneal injection of normal saline (10 g/kg, qd) for 14 d after establishing model	(1) Changes of cardiac pathology (2) MCP-1 mRNA (3) MCP-1	(1) *P* < 0.05 (2) *P* < 0.01 (3) *P* < 0.05

Zhang et al. 2009	Balb/c mice (6-8 weeks; female; 20/20)	NM	By intraperitoneal injection of culture medium containing 10000 TCID_50_ CVB3 virus (0.1 ml)	By intraperitoneal injection of AM injection (0.4 g, qd) for 21 d after establishing model	By intraperitoneal injection of normal saline (0.2 ml, qd) for 21 d after establishing model	(1) Changes of cardiac pathology (2) IL-2 (3) IL-8 (4) IL-18	(1) *P* < 0.05 (2) *P* < 0.01 (3) *P* < 0.01 (4) *P* < 0.05

Li et al. 2007	Balb/c mice (5 weeks; NM; 40/40)	15-16 g	By intraperitoneal injection of culture medium containing 500 TCID_50_ CVB3 virus (0.1 ml)	By oral gavage of AM oral liquid (0.4 g, qd) for 14 d after establishing model	By oral gavage of normal saline (0.3 ml, qd) for 14 d after establishing model	(1) Changes of cardiac pathology (2) Coxsackievirus and adenovirus receptor mRNA	(1) *P* < 0.01 (2) *P* < 0.01

Wu et al. 2007	Balb/c mice (4 weeks; male; 20/20)	14 ± 2 g	By intraperitoneal injection of culture medium containing 100 TCID_50_ CVB3 virus (0.2 ml)	By oral gavage of AM oral liquid (0.2 mg, bid) for 10 d after establishing model	By oral gavage of normal saline (0.2 ml, bid) for 10 d after establishing model	(1) Changes of cardiac pathology (2) TNF-*α* mRNA (3) Survival rate	(1) *P* < 0.05 (2) *P* < 0.05 (3) *P* < 0.05

Yao et al. 2007	Balb/c mice (5 weeks; male; 20/20)	16-20 g	By intraperitoneal injection of culture medium containing 1 × 10^8^ TCID_50_ CVB3 virus (0.1 ml)	By oral gavage of AM oral liquid (30 g/kg, qd) for 5 d after establishing model	By intraperitoneal injection of normal saline (0.1 ml, qd) for 5 d after establishing model	(1) Changes of cardiac pathology (2) TNF-*α*	(1) *P* < 0.05 (2) *P* < 0.05

Chen et al. 2006	Balb/c mice (3 weeks; male; 36/31)	12-15 g	By intraperitoneal injection of culture medium containing 20000 TCID_50_ CVB3 virus (0.1 ml)	By oral gavage of AM oral liquid (2.2 mg/kg, qd) for 7 d after establishing model	By oral gavage of normal distilled water for 7 d after establishing model	(1) Changes of cardiac pathology (2) cTnI (3) SERCA activity (4) ETR maximum binding capacity (5) ETR equilibrium dissociation constant (6) ET-1 (7) ANP (8) Survival rate	(1) *P* < 0.05 (2) *P* < 0.05 (3) *P* < 0.05 (4) *P* < 0.05 (5) *P* < 0.05 (6) *P* < 0.05 (7) *P* < 0.05 (8) *P* < 0.05

Zhang et al. 2006A	Balb/c mice (4 weeks; male; 30/30)	NM	By intraperitoneal injection of culture medium containing 1 × 10^8^ TCID_50_ CVB3 virus (0.1 ml)	By oral gavage of AM granule (30 g/kg, qd) for 5 d after establishing model	By oral gavage of normal saline for 5 d after establishing model	(1) Cardiomyocyte apoptosis rate (2) TNF-*α*	(1) *P* < 0.05 (2) *P* < 0.05

Guan et al. 2005	Balb/c mice (NM; male; 8/6)	17.5 ± 1.2 g	By intraperitoneal injection of culture medium containing 400 TCID_50_ CVB3 virus (0.2 ml)	By intraperitoneal injection of AM injection (90 g/kg, qd) for 9 d after establishing model	By intraperitoneal injection of normal saline for 9 d after establishing model	(1) Survival rate (2) Changes of cardiac pathology (3) AST (4) LDH (5) MDA (6) SOD (7) Affect of electrocardiogram	(1) *P* < 0.01 (2) *P* < 0.05 (3) *P* < 0.05 (4) *P* < 0.05 (5) *P* < 0.05 (6) *P* < 0.05 (7) *P* < 0.05

Liu et al. 2004	Balb/c mice (45 weeks; male; 50/50)	14-16 g	By intraperitoneal injection of culture medium containing 10000 TCID_50_ CVB3 virus (0.2 ml)	By intraperitoneal injection of AM injection (10 g/kg, qd) for 7 d after establishing model	By intraperitoneal injection of phosphate buffered solutions (0.2 ml, qd) for 7 d after establishing model	(1) Survival rate (2) Changes of cardiac pathology	(1) *P* < 0.05 (2) *P* < 0.05

Liu et al. 2003	Balb/c mice (4-6 weeks; male; 12/12)	NM	By intraperitoneal injection of culture medium containing 1 × 10^9^ TCID_50_ CVB3 virus (0.1 ml)	By intraperitoneal injection of AM injection (10 g/kg, qd) for 7 d after establishing model	By intraperitoneal injection of normal saline for 7 d after establishing model	(1) Changes of cardiac pathology (2) Apoptotic index (3) Fas (4) FasL	(1) *P* < 0.05 (2) *P* < 0.01 (3) *P* < 0.01 (4) *P* < 0.05

Li et al. 2003	Balb/c mice (6-8 weeks; male; 20/20)	16-18 g	By intraperitoneal injection of culture medium containing 100 TCID_50_ CVB3 virus (0.1 ml)	By oral gavage of AM oral liquid (0.78 g/kg, qd) for 14 d after establishing model	By oral gavage of normal saline (0.5 ml, qd) for 14 d after establishing model	(1) Changes of cardiac pathology (2) Virus isolation positive rate	(1) *P* < 0.01 (2) *P* > 0.05

Liu et al. 2002	Balb/c mice (NM; male; 10/11)	12.8 ± 1.0 g	By intraperitoneal injection of culture medium containing 9 × 10^9^ PFU/ml CVB3 virus (0.4 ml)	By oral gavage of AM oral liquid (10 g/kg, qd) for 7 d after establishing model	By oral gavage of normal distilled water for 7 d after establishing model	(1) HW/BW ratios (2) Changes of cardiac pathology (3) LDH (4) CK-MB	(1) *P* < 0.05 (2) *P* < 0.05 (3) *P* < 0.01 (4) *P* < 0.05

Note: d: day; AM: Astragalus membranaceus; ANP: atrial natriuretic peptide; AST: aspartate aminotransferase; Bid: bis in die; cTnI: cardiac troponin I; CK-MB: creatine kinase isoenzyme; CVB3: coxsackievirus B3; ET: endothelin; ETR: endothelin receptor; HW/BW: heart weight/body weight; IL: interleukin; LDH: lactic dehydrogenase; MCP: monocyte chemoattractant protein; MDA: malondialdehyde; MIP: macrophage inflammatory protein; NM: not mentioned; PFU: plaque-forming unit; Qd: quaque die; SERCA: sarco endoplasmic reticulum calcium adenosine triphosphatase; Smad: small mothers against decapentaplegic; SOD: superoxide dismutase; TCID50: median tissue culture infective dose; TNF-*α*: tumor necrosis factor-*α*.

**Table 4:** Information of AM of animal study.

Study (years)	Specifications	Source	Concentration (crude drug content)	Quality control reported
Zhang et al. 2017A	Injection	Unknown	Unknown	Unknown
Li et al. 2016B	Granule	Nanjing Tongrentang Pharmaceutical Co., Ltd.	Unknown	Batch number: 140604
Jiang 2013	Injection	Fuda Pharmaceutical Co., Ltd.	2 g/mL	Batch number: 000617
Wang et al. 2011	Injection	Chengdu Di'ao Jiuhong Pharmaceutical Factory	2 g/mL	Traditional Chinese patented medicine WY: Z51021776
Cai et al. 2009	Injection	Chengdu Di'ao Jiuhong Pharmaceutical Factory	2 g/mL	Traditional Chinese patented medicine WY: Z51021776
Zhang et al. 2009	Injection	Hugang Xinya Pharmaceutical Industry (Yangzhou) Co., Ltd.	2 g/mL	Traditional Chinese patented medicine WY: Z32021256
Li et al. 2007	Granule	Baili Pharmaceutical Co., Ltd.	Unknown	Traditional Chinese patented medicine WY: Z20003380
Wu et al. 2007	Oral liquid	Union Hospital affiliated Huazhong University of Science and Technology	1 g/L	Batch number: 020926
Yao et al. 2007	Granule	Baili Pharmaceutical Co., Ltd.	Unknown	Batch number: 030505
Chen et al. 2006	Injection	Fuda Pharmaceutical Co., Ltd.	2 g/mL	Unknown
Zhang et al. 2006A	Granule	Baili Pharmaceutical Co., Ltd.	Unknown	Batch number: 030505
Guan et al. 2005	Injection	Shanghai Tiansheng Pharmaceutical Chemical Industry Research Institute	12 g/mL	Batch number: 20020108
Liu et al. 2004	Injection	Chengdu Di'ao Jiuhong Pharmaceutical Factory	2 g/mL	Unknown
Liu et al. 2003	Injection	Shanghai Hefeng Pharmaceutical Co., Ltd.	2 g/mL	Unknown
Li et al. 2003	Oral liquid	Unknown	2 g/mL	Unknown
Liu et al. 2002	Oral liquid	Unknown	1 g/mL	Unknown

**Table 5:** Risk of bias of clinical studies.

Study	A	B	C	D	E	F	G	Total
Du 2019	**+**	**—**	**—**	**—**	**+**	**—**	**+**	3
Li et al. 2019	**+**	**—**	**—**	**—**	**+**	**—**	**+**	3
Qi et al. 2019	**+**	**—**	**—**	**—**	**+**	**+**	**+**	4
Zhang et al. 2019	**+**	**—**	**—**	**—**	**+**	**—**	**+**	3
Zheng 2019	**+**	**—**	**—**	**—**	**+**	**—**	**+**	3
Liu et al. 2018	**+**	**—**	**—**	**—**	**+**	**+**	**+**	4
Gui 2017	**+**	**—**	**—**	**—**	**+**	**—**	**+**	3
Zhang et al. 2017B	**+**	**—**	**—**	**—**	**+**	**+**	**+**	4
Zhou 2017	**+**	**—**	**—**	**—**	**+**	**—**	**+**	3
Li et al. 2016A	**+**	**—**	**—**	**—**	**+**	**—**	**+**	3
Xing 2016	**+**	**—**	**—**	**—**	**+**	**—**	**+**	3
Wu 2016	**+**	**—**	**—**	**—**	**+**	**—**	**+**	3
Wu et al. 2016	**+**	**—**	**—**	**—**	**+**	**—**	**+**	3
Gao 2015	**+**	**—**	**—**	**—**	**+**	**—**	**+**	3
Lou 2015	**+**	**—**	**—**	**—**	**+**	**—**	**+**	3
Tao 2015	**+**	**—**	**—**	**—**	**+**	**—**	**+**	3
Zhao et al. 2015	**+**	**—**	**—**	**—**	**+**	**—**	**+**	3
Ban 2014	**+**	**—**	**—**	**—**	**+**	**—**	**+**	3
Wang et al. 2014	**+**	**—**	**—**	**—**	**+**	**—**	**+**	3
Liang 2014	**+**	**—**	**—**	**—**	**+**	**—**	**+**	3
Kang 2014	**+**	**—**	**—**	**—**	**+**	**—**	**+**	3
Zhang 2014	**+**	**—**	**—**	**—**	**+**	**—**	**+**	3
Liu et al. 2013	**+**	**—**	**—**	**—**	**+**	**+**	**+**	4
Zhang et al. 2013	**+**	**+**	**+**	**+**	**+**	**+**	**+**	7
Wang 2012	**+**	**—**	**—**	**—**	**+**	**—**	**+**	3
Dong 2011	**+**	**—**	**—**	**—**	**+**	**—**	**+**	3
Hu 2009	**+**	**—**	**—**	**—**	**+**	**—**	**+**	3
Zhang et al. 2006B	**+**	**+**	**—**	**—**	**+**	**—**	**+**	4

Note: A: random sequence generation; B: concealment of allocation; C: blinding of participants and personnel; D: blinding of outcome assessment; E: incomplete outcome data; F: selective reporting; G: other bias; “+” indicates low risk of bias; “-” indicates high risk of bias; and “?” indicates an unclear risk of bias.

**Table 6:** Risk of bias of animal studies.

Study	A	B	C	D	E	F	G	H	I	J	Total
Zhang et al. 2017A	**+**	**—**	**—**	**—**	**—**	**+**	**—**	**+**	**+**	**+**	5
Li et al. 2016B	?	**—**	**—**	**+**	**—**	**+**	**—**	**+**	?	**+**	4
Jiang 2013	**—**	**—**	**—**	**—**	**—**	**—**	**—**	**+**	?	**+**	2
Wang et al. 2011	?	**—**	**—**	**+**	**—**	**—**	**—**	**+**	?	**+**	3
Cai et al. 2009	?	**—**	**—**	**+**	**—**	**—**	**—**	**+**	**+**	**+**	4
Zhang et al. 2009	?	**—**	**—**	**—**	**—**	**—**	**—**	**+**	?	**+**	2
Li et al. 2007	?	**—**	**—**	**—**	**—**	**—**	**—**	**+**	**+**	**+**	3
Wu et al. 2007	?	**—**	**—**	**—**	**—**	**+**	**—**	**+**	?	**+**	3
Yao et al. 2007	?	**—**	**—**	**—**	**—**	**—**	**—**	**+**	?	**+**	2
Chen et al. 2006	**—**	**—**	**—**	**—**	**—**	**—**	**—**	**+**	?	**+**	2
Zhang et al. 2006A	?	**—**	**—**	**—**	**—**	**—**	**—**	**+**	?	**+**	2
Guan et al. 2005	?	**—**	**—**	**—**	**—**	**—**	**—**	**+**	?	**+**	2
Liu et al. 2004	**+**	**—**	**—**	**+**	**—**	**—**	**—**	**+**	?	**+**	4
Liu et al. 2003	?	**+**	**—**	**—**	**—**	**—**	**—**	**+**	**+**	**+**	4
Li et al. 2003	?	**+**	**—**	**—**	**—**	**+**	**—**	**+**	?	**+**	4
Liu et al. 2002	?	**—**	**—**	**—**	**—**	**—**	**—**	**+**	?	**+**	2

Note: A: sequence generation; B: baseline characteristics; C: allocation concealment; D: random housing and animal welfare; E: blinding of caregivers and/or investigators; F: random outcome assessment; G: blinding of outcome assessor; H: complete outcome data; I: selective outcome reporting; J: other sources of bias. “+” indicates low risk of bias; “-” indicates high risk of bias; and “?” indicates an unclear risk of bias.
